# Bottom‐Up Synthesis and Active Assembly of DNA Networks by Biomolecular Nanomachines

**DOI:** 10.1002/smll.202514262

**Published:** 2026-06-11

**Authors:** Farhana Afroze, Richard J Archer, Mahammad Mustakim, Rakesh Das, Arif Md. Rashedul Kabir, Yuuto Miura, Rubaya Rashid, Kazuki Sada, Tetsuya Hiraiwa, Shin‐ichiro M. Nomura, Shogo Hamada, Akira Kakugo

**Affiliations:** ^1^ Department of Chemistry Faculty of Science Hokkaido University Sapporo Japan; ^2^ Department of Robotics Graduate School of Engineering Tohoku University Sendai Japan; ^3^ Department of Computer Science School of Computing Institute of Science Tokyo Yokohama Japan; ^4^ Institute of Physics Academia Sinica Taipei Taiwan; ^5^ Max Planck Institute for the Physics of Complex Systems Dresden Germany; ^6^ Department of Physics and Astronomy Graduate School of Science Kyoto University Kyoto Japan; ^7^ Biomolecular Design Institute CBI Research Institute Tokyo Japan; ^8^ Laboratory for Chemistry and Life Science Dept. Life Science and Technology Institute of Science Tokyo Yokohama Japan; ^9^ Physics Division National Center for Theoretical Sciences Taipei Taiwan; ^10^ Research Center for Autonomous Systems Materialogy Institute of Science Tokyo Yokohama Japan

**Keywords:** active assembly, biomolecular, bottom‐up fabrication, DNA, energy‐dissipative assembly, nanomachines

## Abstract

Active assembly of matter is a defining trait of living systems, enabling the creation of far‐from‐equilibrium materials essential for the functionality of life. This is achieved through energy‐dissipative, multi‐step processes facilitated by biomolecular nanomachines performing bottom‐up chemical and mechanical assembly of matter. Mimicking such active assembly synthetically remains a challenge. Here, a bio‐inspired bottom‐up strategy for energy‐dissipative material assembly, driven by biomolecular nanomachines and overcoming thermodynamic and diffusive constraints, is demonstrated. Specifically, two chemically‐fueled biomolecular nanomachines—DNA polymerase and kinesin—are used to demonstrate a multi‐step chemical synthesis and mechanical manipulation process. This results in a DNA biopolymer network with complex hierarchical morphologies unattainable by self‐assembly alone. DNA polymerase generates DNA, which forms a fibrous 2D‐network when actively connected and pulled between kinesin‐powered motile microtubules. Experimental data and simulations show that both DNA‐DNA interactions and active mechanical forces from molecular motors are essential to this process. Furthermore, key factors for network formation are investigated by systematically investigating DNA polymerase incubation time and microtubule density. The present work provides a key step toward bottom‐up fabrication of complex and dynamic materials by mimicking the sophisticated assembly strategies of living systems, potentially providing a framework for future materials assembled by nanomachines.

## Graphical abstract

This study demonstrates a dynamic, bottom‐up assembly of DNA materials driven by two biomolecular nanomachines: DNA polymerase and kinesin. Through sequential chemical synthesis and motor‐driven mechanical manipulation, DNA fibrous networks are dynamically formed with complex morphologies unattainable by self‐assembly alone. The work demonstrates a step toward a bio‐inspired platform for constructing active, far‐from‐equilibrium materials.

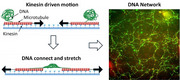

## 1 Introduction

Biomolecular nanomachines lie at the core of life's capacity to generate and maintain dynamic materials. These specialized molecules, including enzymes and molecular motors, harness energy from chemical reactions to perform mechanical and chemical work. Through energy dissipative processes, they actively assemble and organize molecular components into complex, hierarchical structures with spatiotemporal control [1, 2, 3, 4, 5, 6]. Harnessing such nanomachines could be key to synthetically constructing far‐from‐equilibrium materials that mimic the structural and functional sophistication of living systems.

The remarkable operations and efficiencies of these natural nanomachines have created an ever‐growing demand for their utilization in industrial processes for the synthesis of complex biologically active molecules [7, 8, 9], as well as clinical and laboratory applications [10, 11]. With recent developments in biochemistry and chemistry improving accessibility to biomolecules, chemical systems are also more frequently utilizing biomolecular agents derived from nature for the synthesis and morphology control of macromolecular structures [12, 13, 14, 15, 16]. Notably, active transport using protein‐based molecular motors is gaining interest as a potential method to overcome the limitations of diffusion in bottom‐up nanoscale fabrication [17, 18, 19]. Biomolecular nanomachines are highly specialized with singular functionalities performed with high activity and efficiency. The complexity of natural systems arises from employing synergistic combinations of active nanomachines. Multiple molecular nanomachines, each contributing to sequential energy‐consuming steps, are able to work together to produce ordered structures which could not be formed through single‐step or non‐energy‐consuming processes alone [20, 21, 22]. For instance, cellular cytoskeletons, a vital component involved in the control of shape and motility of cells, require a multitude of active nanomachines facilitating energetic steps, including protein synthesis, molecular transport, and mechanical deformations [23, 24, 25, 26].

Although the synthetic, dynamic generation of hierarchical materials through dissipative chemical processes has been documented, up to present, it has only been achieved through chemical synthesis, however, without energy dissipative assembly [12, 27, 28, 29, 30, 31]. Taking inspiration from natural systems, we hypothesize that combining multiple classes of energy‐dissipative biomolecular active nanomachines may enable a multistep process for both molecular synthesis and subsequent mechanical assembly into hierarchical structures.

Specifically, we demonstrate the dynamic generation of 2‐D DNA networks through a sequential two‐step process. First, DNA polymerase synthesizes long DNA macromolecules via rolling circle amplification (RCA) of DNA templates tethered to microtubules. Subsequently, kinesin motor proteins generate mechanical forces that actively stretch and organize these synthesized DNA strands into fibrous, hierarchical network structures. Coarse‐grained simulation results supported the hypothesis that active motion (self‐propulsion) can overcome equilibrium state forces to produce stretched network structures. Acting together, through multi‐step energetic operations, this molecular system was shown to be able to generate DNA networks in a dynamic fashion through chemical energy consumption.

While other systems, such as RCA, have been widely used to generate DNA networks and motor proteins have been extensively studied in active matter systems, these approaches have typically been used independently, resulting in isotropic network formation or individual filament formation without higher‐order structural patterning. Here, by coupling in situ DNA growth with kinesin‐driven microtubule transport, motor activity directly organizes and stretches the growing DNA into extended network architectures. This enables active control over network formation and spatial organization, allowing the formation of dynamically patterned DNA materials. Unlike systems such as DASH, this approach does not require prefabricated microfluidic architectures, therefore providing a versatile route toward actively organized biomolecular materials and offering a step toward a new experimental framework for studying force‐driven assembly in active matter systems [12].

## 2 Results and Discussion

**FIGURE 1:**
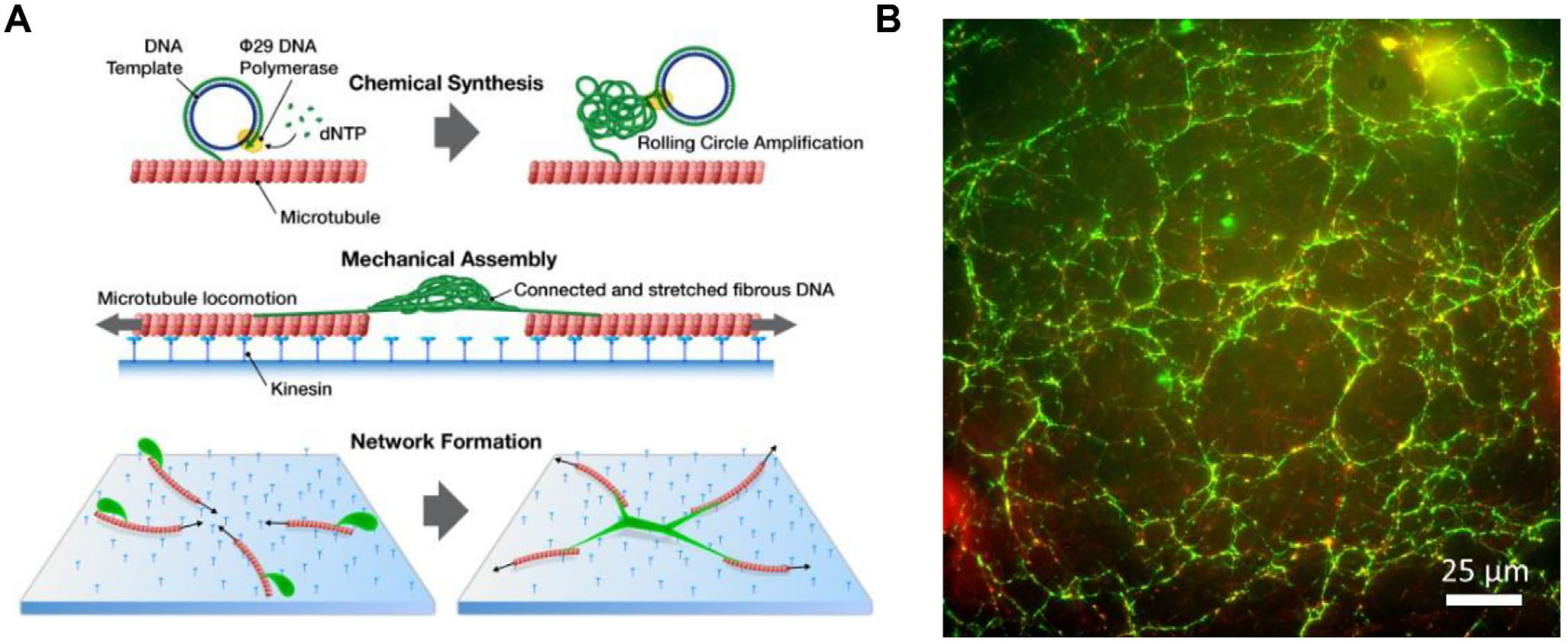
DNA hierarchical network formation (A) Schematic presentation of 2‐D DNA network formation by polymerase amplification of RCA template modified microtubules (chemical synthesis), and kinesin motor protein facilitated stretching of RCA‐amplified DNA (mechanical assembly), leading to network formation by collisions of DNA modified microtubules. (B) Experimentally observed DNA (green) network with microtubules (red).

**FIGURE 2:**
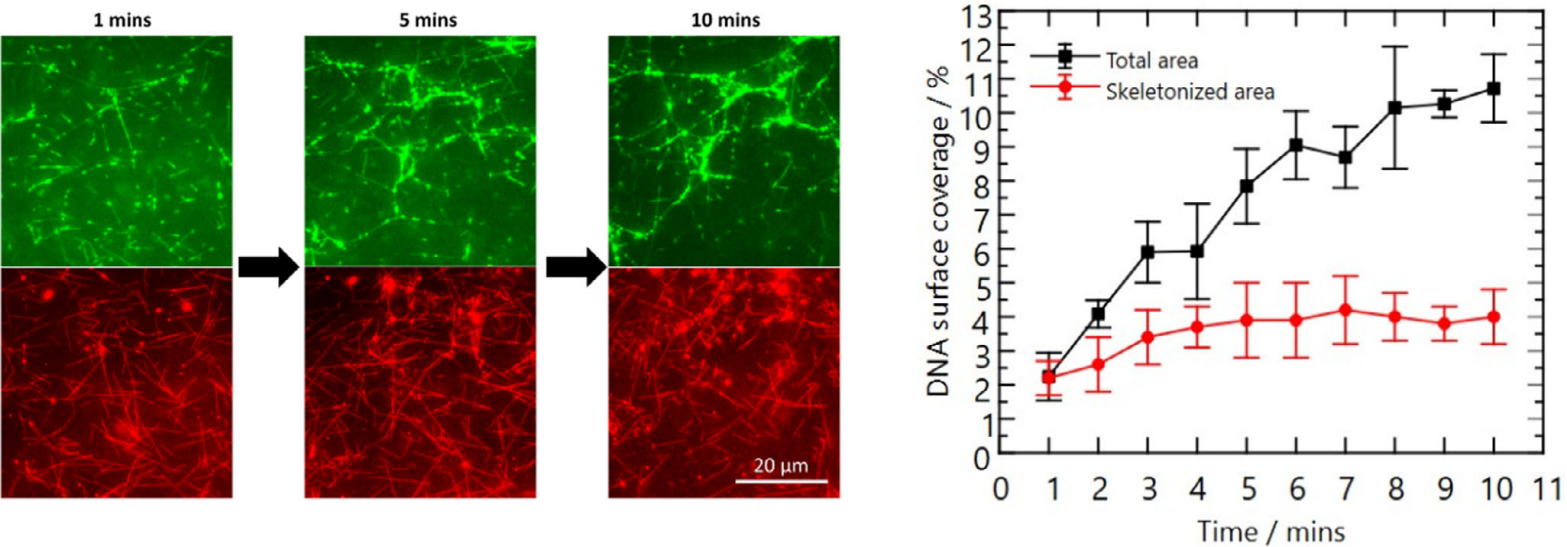
(A) Fluorescence microscopy images of the DNA network formation process. Images were taken immediately after injection (1 min) into the flow cell and after 5 and 10 min of the microtubule motility, left and right, respectively. DNA is presented in green, and microtubules in red. (B) Graph showing the percentage surface coverage of DNA as a function of total area (All visible DNA) vs skeletonized (central line) area.

Full Experimental methodology is given in full in the Supporting Information. Briefly, DNA‐template tethered microtubules were produced through click chemistry between azide‐labeled microtubules and the primer DNA hybridized to the template DNA, as described by existing protocols [12, 32, 33, 34]. DNA‐ligated microtubules were washed to remove unbound DNA, and then incubated for 1–10 h with Φ29 DNA polymerase. The reaction extends the single‐stranded DNA tethered to microtubules through nucleotide addition, increasing strand length without increasing the number of DNA attachment points.″ To observe the mechanical effect of motile microtubules stretching the DNA, first, a flow cell coated by motor proteins was prepared by following a typical microtubule gliding assay protocols [35, 36, 37]. DNA‐tethered microtubules were then introduced into the modified flow cell and incubated for 4–10 min to allow attachment to the surface. Finally, a buffer containing ATP was injected through the flow cell, and observations were started immediately after. The motion of the DNA‐tethered microtubules due to the ATP‐powered kinesin was found to move in random directions, equivalent to a typical microtubule motion without modification (Movie S1). Pre‐synthesized DNA was observed to become stretched between the tethered microtubule and the surface or between two tethered microtubules (Movies S2 and S3). The random motion of multiple microtubules stretching multiple DNA strands created a network structure to develop when the observable DNA fibers were pulled into contact with each other (Figure 1A,B; Movie S4). The patterned structures were dynamically generated on the glass interface over 10 min of ATP introduction into the flow cell. While the DNA formed clear interconnected structures, the microtubules showed a much weaker correlation to the DNA network. As the overlay between DNA and microtubules was not a perfect match, we conclude that this is not simply an overlay pattern caused by DNA‐tethered microtubules, but is likely formed by the physical manipulation of the DNA by active nanomachines. (Figure 2). Figure 2B shows that DNA surface coverage increases over time. The skeletonized area (representing network paths without regard for thickness) increases during the first 4 min and subsequently plateaus, whereas the total area continues to increase up to 10 min. This indicates that new network paths are established during the initial phase, followed by continued thickening of existing structures. Tortuosity analysis during the initial stages of network formation (1, 5, and 10 min) showed values consistently close to 1 (1.10, 1.12, and 1.14), with no significant deviation over time. This indicates that the increasing network density arises primarily through the addition of straight connections, consistent with the incorporation of mechanically stretched DNA strands rather than the formation of a relaxed structure.

**FIGURE 3:**
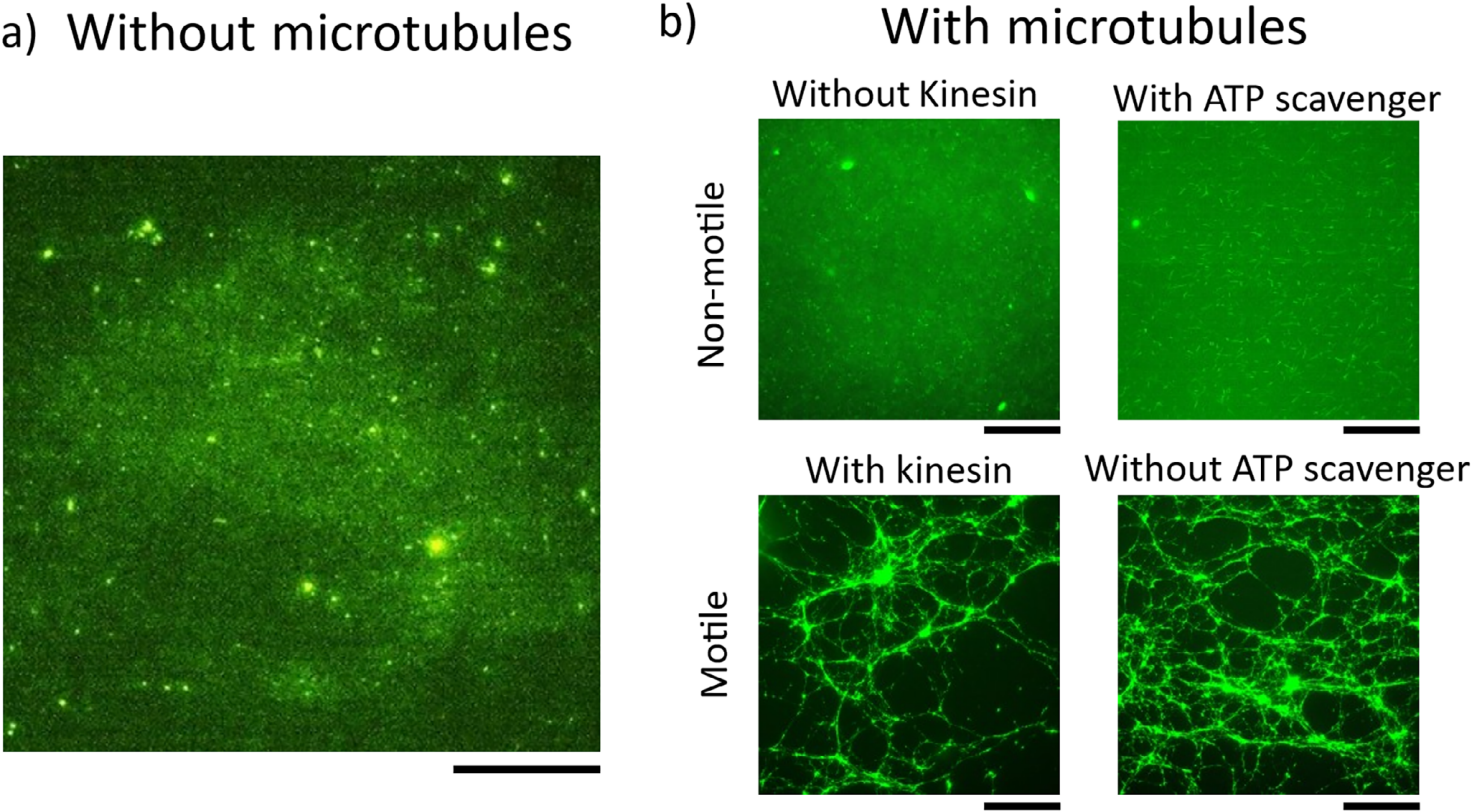
Active assembly control studies, images are representative of multiple independent experiments. (A) Fluorescence microscopy images of DNA network formation assay in the without microtubules (B). Fluorescence microscopy images of DNA network formation with microtubules either in a non‐motile (top row) or motile state (bottom row). Microtubule motility was controlled by either the absence or presence of kinesin (left) or by adding an ATP scavenger (right). Scale bars represent 20 µm.

The motility of DNA‐tethered microtubules and their ability to do work are therefore considered a key factor in DNA network formation. In order to verify the effect of motility, DNA‐tethered microtubules were injected into flow cells with or without kinesin modification. Without kinesin, the microtubules did not adhere to the glass surface, and no DNA networks were observed (Figure 3A). Kinesin‐modified flow cells, on the other hand, reliably formed DNA networks on the surface (Figure 3B). Further, to show that the kinesin must also be kinetically active and not simply act as a surface linker, DNA network formation was tested in kinesin‐modified flow cells with and without ATP fuel. Apyrase, an ATP scavenger, was also added to the cells without ATP to ensure no residual fuel remained from the DNA‐microtubule preparation. Microtubules would be expected to bind to the surface in both cases, but only show motility in the presence of ATP. As expected, in cases with ATP present and no apyrase included, DNA network formation occurred. Without ATP (and with the addition of ATP‐depleting apyrase), no microtubule motion was detected, and subsequently, no DNA network formation was observed (Figure 3B). From these results, we have shown that the active motility of the DNA‐tethered microtubules is critical in order to form DNA networks. While the energy balance cannot be quantitatively determined in the present system, the results suggest that the material forms through an energy‐dissipative process driven by ATP hydrolysis, where kinesin motor activity generates forces that move microtubules and mechanically stretch the attached DNA.

**FIGURE 4:**
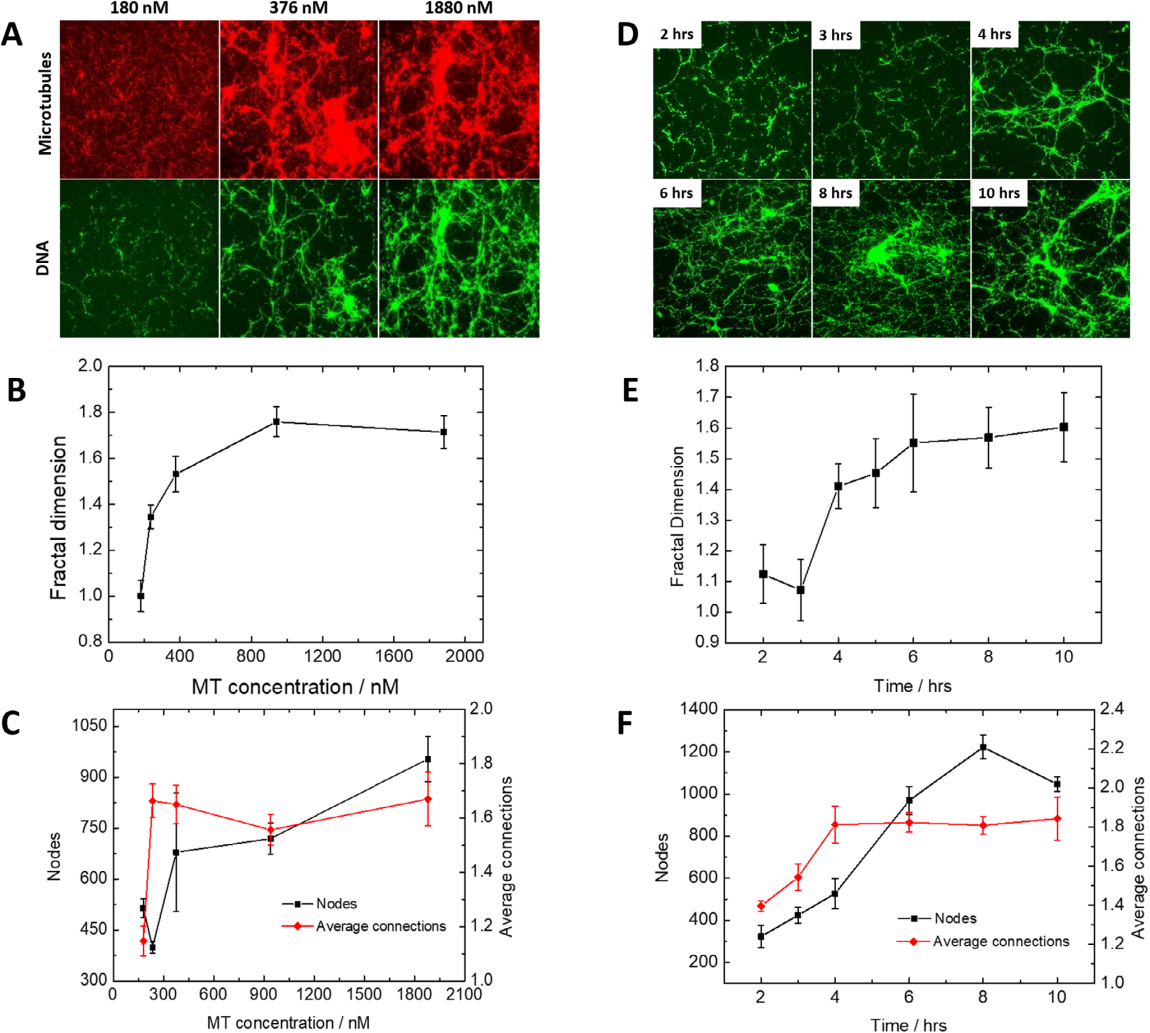
Conditions for DNA network formation (A) Representative fluorescence images of different concentrations of amplified DNA conjugated microtubules (Top, red) and amplified DNA (Bottom, green) on a kinesin‐coated glass surface. (B) Graph showing the calculated average fractal dimension (FD) of the DNA network at different concentrations of DNA‐conjugated microtubules (n = 3). (C) Graph showing the calculated average number of nodes (black squares) and their average number of connections (red diamonds) at different concentrations of DNA‐conjugated microtubules (n = 3). (D) Representative fluorescence images of DNA network formation on the kinesin‐coated glass surface after 2, 3, 4, 6, 8,, and 10 h of pre‐incubating the DNA‐tethered microtubules with the RCA amplification reagents in a tube. (E) Graph showing the calculated average fractal dimension of the DNA network that is formed with different incubation times of DNA‐tethered microtubules with amplification reagents (n = 3). (F) Graph analysis of the calculated average number of nodes (black squares) and their average number of connections (red diamonds) at different incubation times of DNA‐tethered microtubules with amplification reagent (n = 3).

For the formation of any network, a critical density of the material is required where the likelihood of contact and connection between the observable individual units becomes a statistical certainty. In the case of microtubule‐mediated DNA networks, both microtubule density and DNA density can be considered as essential variables for successful network formation. By varying the concentration of tubulin in the microtubule preparation, the resulting DNA‐microtubule density could be controlled. Figure 4A shows representative results of DNA network formation (green) using DNA‐tethered microtubules assembled from different tubulin concentrations (red) at a constant DNA synthesis time (6 h). Microtubules across all samples were found to be 7.4 ± 2.1 µm, with the main difference being the number of microtubules in the sample, increasing relative to tubulin concentration. Microtubules formed with 180 nm of tubulin showed a moderate density of microtubules in the flow cell. However, although some separated fibrous structures can be observed, the density was too low for the DNA to be stretched long enough for network formation. As the density of microtubules increased (tubulin 367 nm), a more connected DNA network was formed. The connection of the network seemingly continues to increase with higher microtubule density. At a tubulin concentration of 1880 nm the DNA forms a highly connected network. The morphology of networks was assessed quantitatively by using fractal dimension (FD), a widely known method to quantify the complexity of networks [38]. In addition, graph theory was also used to analyze the connectivity by identifying nodes (significant clusters of pixels) and their average connections (number of connecting edges between nodes) [39]. Figure 4B shows the FD against microtubule density; the increase in the FD suggests that the DNA network showed an increase in structural complexity as the microtubule density increased. This occurs as a large jump from 180 to 235 nm and a smaller increase from 235 to 1880 nm. Figure 4C shows the number of nodes (black squares) increasing slowly with increasing microtubule concentration; however, the number of connections (red diamonds) between the nodes showed a sharp increase from 180 to 235 nm but reached a plateau at higher concentrations, reaching a limit of approximately 1.6 connections per node. The jump in both FD and the number of connections indicated the critical transition point in the network formation depending on the microtubule concentration. In addition, while the total DNA quantity increases correlated to the DNA‐microtubule concentration, the microtubule attachment in the microfluidic cell depends on a surface area‐limited interface, therefore restricting the maximum number of DNA‐tethered microtubules that can interact with motor proteins, resulting in a limit in the node value.

The effect of the length of DNA on the resulting network structure was investigated by varying the incubation time for DNA synthesis. Although direct measurement of DNA length is not feasible, longer incubation times are expected to produce proportionally longer DNA strands. Figure 4D shows the resulting DNA network formation at select incubation times. After 2 to 3 h of incubation, DNA formed fibrous structures but with low connectivity. Increasing the incubation time to 4 h resulted in well‐connected network structures. From 4 to 8 h, the increasing incubation time visibly seems to increase the connectivity and density of the formed DNA structure; however, further increasing the incubation time to 8–10 h seems to have less effect on the resulting structure. Figure 4E shows the change in FD of the DNA network with the incubation time. Generally, as incubation time increases, the FD increases, suggesting a growing complexity in the structure. Interestingly, this increase in FD does not happen linearly; however, a sudden increase occurs at 3 to 4 h, suggesting the length of DNA reached a critical concentration between these times, which allowed it to form a well‐connected structure. Figure 4F shows the number of nodes (black squares) increasing in a linear fashion up to 8 h of incubation and a slight decrease from 8 to 10 h. The average number of connections (red diamonds) for each node, however, increases up to 4 h of incubation and plateaus afterward at an average of 1.8 connections per node. This may indicate an upper limit to the number of connections that can be made, even if the length of DNA increases. These observations suggest that network formation depends on microtubule density, DNA concentration, and DNA length. In the present system, these parameters are indirectly controlled through tubulin concentration and RCA incubation time, which modulate filament density and effective DNA length, respectively. The results indicate that network formation occurs once sufficient filament density and DNA extension are achieved, rather than following a simple linear relationship.

**FIGURE 5:**
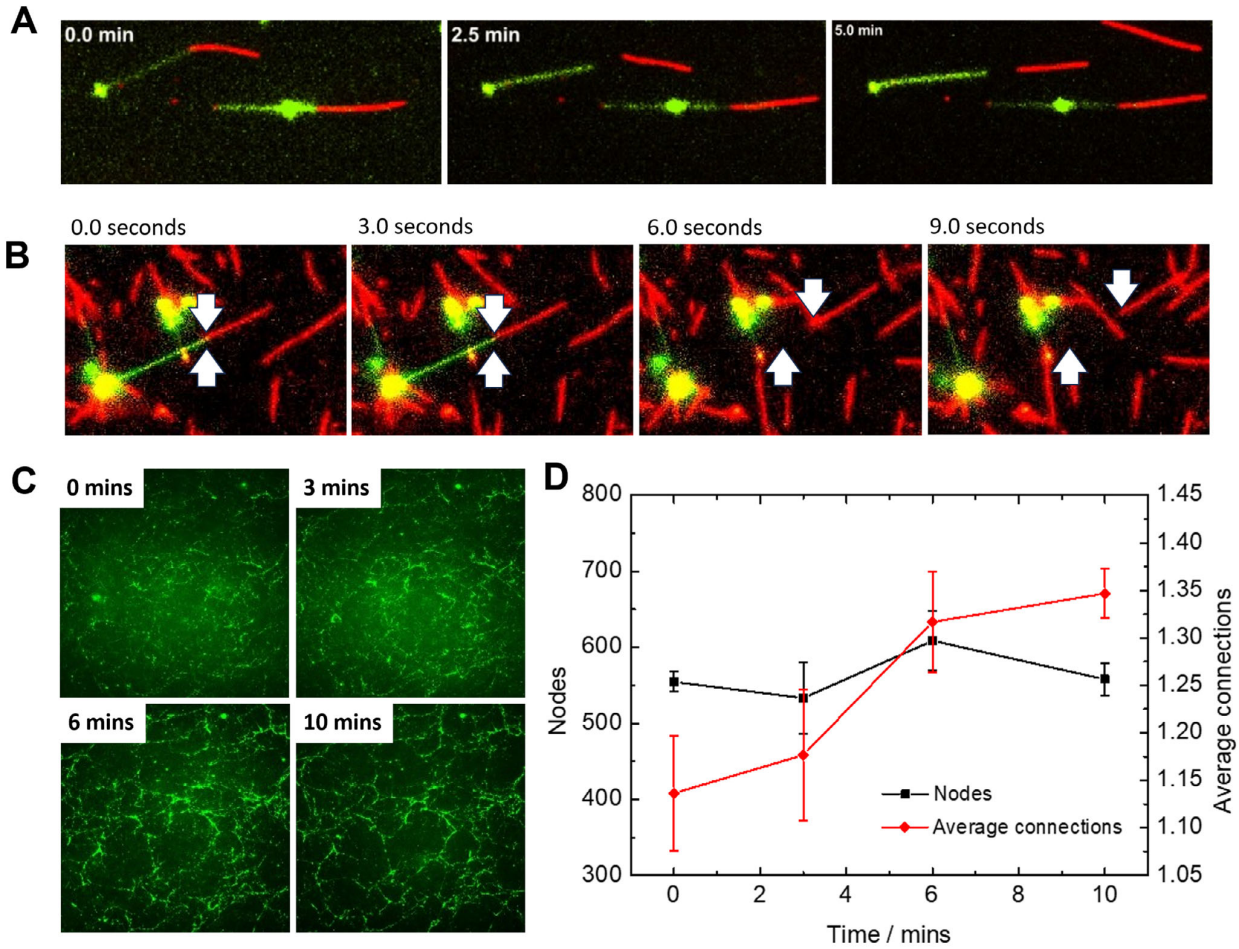
Observed DNA network formation (A) Observed stretching of individual DNA‐tethered microtubules. DNA (green) caught between the surface and the motile microtubules (red) was observed over a period of 5 min. Scale bar: 5µm. (B) Observed breaking of stretched DNA and loss of fibrous morphology, top arrow indicates the trailing end of the microtubule, and the bottom arrow indicates the observed maximum extended position DNA fiber before breaking (C) Time‐lapse observation of DNA network formation over time after initial injection into the kinesin‐coated flow cell (0 mins), and at further 3, 6, and 10 min observations. (D) Graph analysis of time‐lapse observation showing the calculated number of nodes (black square) and the average number of connections between them (red diamond) during the DNA network formation.

To investigate the detailed mechanisms behind the DNA network formation, the individual stretching behavior of DNA‐tethered microtubules in our system was observed. Similar phenomena have also been reported elsewhere with DNA bridged to microtubules through streptavidin‐biotin links [40, 41, 42, 43]. The observed behavior confirmed that active stretching of DNA directly covalently linked to microtubules occurs during the assay and confirms a critical function for the formation of the fibrous structures constituting links of the network (Figure 5A; Movies S2, S3, and S5). The critical interactions for DNA stretching appear to be through DNA‐to‐DNA interactions (Movies S2, S3, and S5), whereas microtubule‐to‐microtubule interactions and microtubule‐to‐DNA interactions appeared to be negligible. It can be observed that gliding microtubules without observable attached DNA move past one another or across DNA without measurable changes in direction or velocity, indicating minimal interaction. (Movies S1–S6, respectively). Interestingly, DNA fibers have been observed to occasionally break, leading to the rapid loss of their stretched fibrous structure (under 3 s), showing that the fibrous structures are not naturally in thermodynamic equilibrium. At maximal DNA extension, the opposing forces from microtubule motion and DNA elasticity are seemingly balanced, resulting in no net movement. Upon DNA fiber breaking, the microtubules immediately resume motion (Figure 5B; Movie S5). These observations indicate that, although the DNA network appears structurally stable on the timescale of observation, motor‐driven forces likely play an important role in generating network formation and may also be needed for maintaining DNA in a stretched configuration. The resulting architecture is therefore consistent with an energy‐dissipative assembly process, although further experiments would be required to determine whether continued energy consumption is necessary to maintain the network structure. Time‐lapse observation of DNA network formation revealed that the formation dynamically occurred within minutes after being injected into kinesin‐modified flow cells (Movie S4). Initial observations straight after injection into the flow cell (0 min) showed some small fibrous structures, but with low connectivity. After 3 min, the number of connections appears to increase slightly, and after 6–10 min, the network has largely formed with strong fibrous structures connecting across the observed area (Figure 5C). Several contributing mechanisms were observed in forming the final network in addition to the DNA stretching, including DNA tethered microtubule‐induced rearrangement of the DNA fibers (Movie S2), DNA addition from the bulk to the surface (Movie S7), and DNA fiber thickening by DNA fiber‐to‐fiber attraction and aggregation (Movie S8). Quantitative assessment by graph analysis showed that the number of nodes does not significantly change over 10 min, while the number of average connections (edges per node) increased steadily (Figure 5D). This suggests that the quantity of the DNA is not significantly changing over the observation time, but the interconnection of the DNA (observed as edges) occurred at a constant rate due to the linear stretching of observable individual DNA‐tethered microtubules over this time.

**FIGURE 6:**
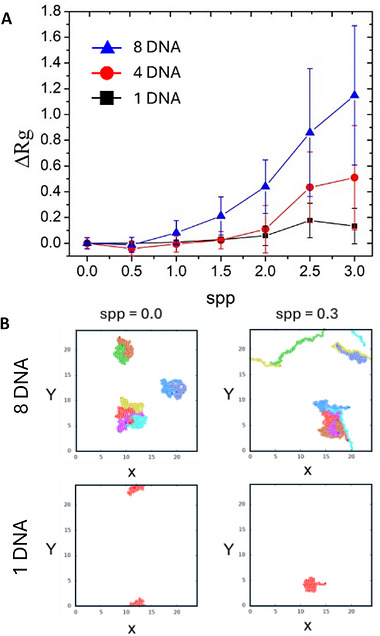
coarse‐grained simulation results showing (A) DNA analogues with 1, 4, or 8 simulated units, average change in the radius of gyration (ΔRg) against an applied self‐propulsion force (SPP) (n = 3) (B) Example x, y plots from simulation results showing 1 or 8 DNA analogues (DNA) against Brownian motion without (SPP = 0.0) or with self‐propulsion.

Finally, coarse‐grained simulations are used as a minimal conceptual framework to explore how active propulsion and filament interactions can drive the emergence of extended network architectures, following established active matter models of interacting self‐propelled particles [44]. While not intended to quantitatively reproduce the experimental system, the simulations provide qualitative support for the proposed mechanism by demonstrating that certain key factors, such as active propulsion, can drive the emergence of extended and connected network structures. Full details of the simulation are given in the Supporting Information. Briefly, the abstract DNA model with interactions mimicking hydrogen bonding was given by a spring ball model with 64 monomer chains (representing nucleotides) with Lennard‐Jones potential interactions. Active motions of the microtubules were simulated by a self‐propulsion force (SPP) applied to one terminal monomer on each chain. The change in the radius of gyration (ΔRg) was measured as a quantitative metric to characterize the anisotropic formation against SPP (Figure 6A).

Under Brownian motion without a self‐propulsion force (SPP = 0.0), DNA‐DNA interactions were found to be the dominating factor causing each polymer to fold into aggregated thermodynamic minimal states with low R_g_, regardless of the number of DNA analogues. Interestingly, when SPP was increased up to SPP = 0.3, R_g_ did not significantly increase for cases of only 1 DNA but increased significantly when simulated with 4 or 8 DNA analogues together (corresponding to higher concentration in the experiments, Figure 4; Movies S9 and S10). Positional plots indeed showed the connected anisotropic formation of chains at higher SPP values with 8 DNA analogues (Figure 6B). At SPP values above 0.3, even single 1 DNA analogues unfold, leading to an increase in R_g_. In these cases, the results do not correspond well with the experimental observations; therefore, we focused on only the range of SPP equal to or smaller than 0.3.

These results suggest that combinations of directional self‐propulsion forces competing with DNA self‐interactions led to the unfolding and connection of the chains. The increase in R_g_ depending on the number of DNA analogues also corresponded well to qualitative tendencies observed in experimental results by varying the concentration. In conclusion, the simulation results supported the proposed mechanism behind this fibrous network assembly behavior as an energy‐dissipative process; that is, a balance of active motile forces and DNA‐DNA interactions is necessary to create these structures.

## 3 Conclusion

Here, we demonstrated the use of multi‐step acting biomolecular nanomachines as the central drivers in the dynamic formation of 2‐D DNA hierarchical network structures. Two distinct classes of active biomolecular nanomachines, DNA polymerase and kinesin motor proteins, were employed to construct a dynamic biomaterial system. Microtubules with tethered DNA primers were utilized both as a starting point of DNA synthesis by RCA reactions using DNA polymerase, as well as mediators in generating forces through kinesin motor proteins, which played as an active agent of the system. ATP was found to be necessary for the system, demonstrating that active motion of such nanomachines was a critical factor in the dynamic material generation. Via the random gliding motion of DNA‐tethered microtubules, extended DNA was stretched and pulled together into micron‐scale, observable, interconnected network structures. Parameters such as the total amount and length of DNA and the density of microtubules were experimentally investigated to understand the critical conditions for network formation. The formation has also been observed by time‐lapse imaging, showing that the process dynamically occurred within minutes after introducing DNA‐tethered microtubule units into kinesin‐coated microfluidic flow cells. Supported by coarse‐grained simulations, DNA network formation is achieved through a complex balance of forces between active motile forces competing with DNA‐DNA interactions to form actively manipulated structures. While demonstrated here in the specific Φ29 DNA polymerase and kinesin–microtubule system, these results represent steps toward a model platform for investigating how multiple active biomolecular processes can cooperate to generate higher‐order structure.

The present results provide consistent support for a motor‐driven mechanism of network formation, suggested by multiple structural analyses. While the mechanistic interpretation is primarily inferred from these descriptors, and direct quantitative correlation with independently measurable experimental parameters remains to be established, the observed dependence of network formation on ATP‐driven activity suggests a dynamic assembly process, however the role of energy dissipation remains suggestive. Future work will aim to establish quantitative links between the microtubule activity and emergent network properties, enabling a more rigorous mechanistic description of the system.

Conceptually, our work demonstrates a step toward a potential strategy for assembling out‐of‐equilibrium biomaterials by utilizing active molecular nanomachines. Such types of systems could be developed as a platform to develop bio‐inspired materials with dynamic properties, such as self‐regeneration. We believe this approach could open new avenues toward developing synthetic biomolecular materials with plasticity, demonstrating adaptive or responsive behaviors [45]. Toward acting as a model system, this platform may also contribute to understanding composite cytoskeletal networks by enabling controlled investigation of how motor‐generated forces drive network assembly and spatial organization. The ability to harness active transport to reorganize molecular components into extended, connected architectures provides insight into how force generation can regulate network connectivity, pattern formation, and emergent material structure in active biomolecular systems. Previous studies of active composite cytoskeletal networks have primarily focused on microtubule–actin systems, which can undergo dynamic restructuring under kinesin‐driven force generation [46, 47, 48, 49]. However, these systems are mechanistically complex, involving a large number of interacting proteins with coupled biochemical and mechanical roles. The present system offers a complementary route in which motor‐generated forces actively form network architecture by stretching and connecting a polymer during assembly. In this DNA–microtubule composite, flexible DNA strands act as extensible connectors that are stretched by motor‐driven microtubule motion, while the microtubules themselves remain largely unaffected by the DNA structures. This behavior contrasts with actin–microtubule composites, where bidirectional coupling between components can arise through crosslinking or depletion‐mediated interactions. Importantly, DNA provides an additional level of tunability compared with cytoskeletal filaments, as sequence design allows interactions and connectivity to be programmed at the molecular level. Together, these features suggest that the system provides a controllable model for studying force–architecture coupling in active biomolecular materials [19, 50]. To develop this system as a model, future studies will focus on further investigating the mechanistic basis of network formation and aim to establish a clear quantitative link between experiments and modeling. More broadly, the demonstration of mechanical material assembly by active nanomachines holds significant implications for bottom‐up fabrication in nanotechnology. As the synthesis and control of motile artificial nanomachines become increasingly feasible, the prospect of harnessing controlled energy‐dissipative processes for the programmable nanofabrication of complex materials from the molecular scale upward is becoming ever more attainable [51, 52, 53, 54].

## Funding

This work was supported by JSPS Grants‐in‐Aid for Scientific Research (22K12239, 22H05396, 20H05969, 25K22239, 25K21817, 25K01194, 26H02543, 26H00482 for S.H.; JP21H04434, JP18H05423, 25H00608, 26H00387, for A.K.), JST PRESTO (JPMJPR23Q7 for S.H.), the Futaba Foundation (S.H.), Japan Prize Foundation (Heisei Memorial Research Grant) (S.H.), Japan Association for Chemical Innovation (S.H.), the New Energy and Industrial Technology Development Organization (JPNP20006 for A.K.), and National Science and Technology Council, Taiwan (NSTC113‐2112‐M‐001‐426051‐MY3 for T.H.).

## Conflicts of Interest

The authors declare no conflicts of interest.

## Supporting information




**Supporting File 1**: smll73489‐sup‐0001‐SuppMat.docx.

**Supporting File 2**: smll73489‐sup‐0002‐Movie S1.mp4.

**Supporting File 3**: smll73489‐sup‐0003‐Movie S2.mp4.

**Supporting File 4**: smll73489‐sup‐0004‐Movie S3.mp4.

**Supporting File 5**: smll73489‐sup‐0005‐Movie S4.mp4.

**Supporting File 6**: smll73489‐sup‐0006‐Movie S5.mp4.

**Supporting File 7**: smll73489‐sup‐0007‐Movie S6.mp4.

**Supporting File 8**: smll73489‐sup‐0008‐Movie S7.mp4.

**Supporting File 9**: smll73489‐sup‐0009‐Movie S8.mp4.

**Supporting File 10**: smll73489‐sup‐0010‐Movie S9.avi.

**Supporting File 11**: smll73489‐sup‐0011‐Movie S10.avi.

## Data Availability

The data that support the findings of this study are available from the corresponding author upon reasonable request.
