# Supplementary material for: Bottom‐Up Synthesis and Active Assembly of DNA Networks by Biomolecular Nanomachines
Source: Small. 2026 Jun 11;22(36):e14262. doi: 10.1002/smll.202514262 (PMC13306918; doi:10.1002/smll.202514262)
Supplement: Supplementary file 1 — Supporting File 1: smll73489‐sup‐0001‐SuppMat.docx. [file SMLL-22-e14262-s002.docx]

Supporting Information

**Bottom-Up Synthesis and Active Assembly of DNA Networks by Biomolecular Nanomachines**

Farhana Afroze^1^, Richard J Archer^2,6^, Mahammad Mustakim^3^, Rakesh Das^4^, Arif Md. Rashedul Kabir^1^, Yuuto Miura^1^, Rubaya Rashid^5^, Tetsuya Hiraiwa^3^, Shin-ichiro M. Nomura^2^, Shogo Hamada^6,7*,^ Akira Kakugo^1,5*^

^1^Department of Chemistry, Faculty of Science, Hokkaido University, Sapporo, Japan.

^2^Department of Robotics, Graduate School of Engineering, Tohoku University, Sendai, Japan.

^3^Institute of Physics, Academia Sinica, Taipei, Taiwan

^4^Max Planck Institute for the Physics of Complex Systems, 01187, Dresden, Germany

^5^Department of Physics and Astronomy, Graduate School of Science, Kyoto University, Kyoto, Japan.

^6^Department of Computer Science, School of Computing, Institute of Science Tokyo, Yokohama, Japan.

^7^Biomolecular Design Institute, CBI Research Institute, Tokyo, Japan

**The SI includes:**

Experimental method

Mathematical models and further simulation details.

Figures. S1 to S3

**Supporting movies for this manuscript include the following:**

Movies. S1 to S8

**1. Experimental section**

*1.1 Purification of tubulin and kinesin and labelling of tubulin*

A high-concentration PIPES buffer (1 M PIPES, 20 mM EGTA, and 10 mM MgCl2; pH adjusted to 6.8 using KOH) was used to purify tubulin from the porcine brain. To prepare high-concentration PIPES buffer and 80 mM PIPES buffer (BRB80), PIPES from Sigma was used, and KOH was used to adjust the pH.^25^ Recombinant kinesin-1 consisting of the first 573 amino-acid residues of human kinesin-1 was prepared by following the purification method in the literature.^26^ ATTO-565 labelled tubulin was prepared using ATTO-565 succinimidyl ester (ATTO-565; ATTO-Tec Gmbh) according to the standard techniques.^27^ The labelling ratio of ATTO-565 labelled tubulin was 1.0. The ratio was determined by measuring the absorbance of tubulin and ATTO-565 dye at 280 nm and 564 nm respectively. The labelled tubulin was diluted with BRB80 buffer that the concentration of tubulin in the solution became 70 µM.

*DNA sequence and template circularization process*

The sequences of both primer and template DNA^7^ are given below:

Primer: /5DBCOTEG/GACCACCTTCGCGTCCAAAGC >

Template:/5Phos/CGAAGGTGGTCTTTTTTTTTATATAGAATTCTATATATTTTTTTTGCTTTGGACG >

Sequences are written with 5’-3’ direction. Blue and Red pairs represent complementary sequences. To form a circular template DNA, the template and primer DNA were mixed with equimolar ratio in 1× phi29 DNA polymerase reaction buffer (ThermoFisher Scientific, USA) (8 µM and 8 µL of each DNA) and then annealed down from 95°C to 4°C (−1°C/min) by thermal cycler. 4 µL T4 DNA Ligase (400U) and 6 µL ATP (2.5 mM) were added to hybridized solution (4 µM) and incubated at 4°C for overnight for the ligation (circularization of Template DNA). After ligation, final concentration of DNA became 2 µM.

*1.2 Preparation of DNA conjugated microtubules*

microtubules were polymerized in a tube containing 3.2 μl azide and 0.8 μl ATTO-565 tubulin. 1 μl polymerization buffer (80 mM PIPES, 1 mM EGTA, 5 mM MgCl_2_, 1 mM GMPCPP; pH 6.8) was added to the tubulin mixture and incubated in a water bath at 37 ^o^C for 30 minutes. The prepared azide labelled microtubules were then stabilized adding 0.5 μl taxol (1 mM). Azide Alkyne cycloaddition reaction (click reaction) was performed in the next step to modify the microtubules with DNA. 5 µl azide labelled microtubules, 0.5 µl taxol were mixed with 3.5µl ligated DNA and 1 µl 4×BRB80. This mixture was then incubated at 37 ^o^C for 6 hours. After the incubation, free DNA from the samples of DNA modified microtubules was removed by centrifugation. The solution was added carefully into 100μl Cushion buffer and centrifuged at 54000 rpm for 1 hr at 37 ^o^C. The supernatant was removed from the top and the pellet was washed with 100 μl taxol buffer. The pellet was then dissolved gently using 15 μl taxol buffer. The final stock of ligated DNA modified microtubules was prepared.

*1.3 Amplification of ligated DNA conjugated microtubules by rolling circle amplification (RCA)*

Ligated DNA modified microtubules were used as a template in RCA reaction. Ligated DNA solution was then mixed with 1mM dNTP (dATP, dGTP, dTTP, dCTP), SYBR Green I, and 5U/µL Φ29 DNA polymerase in 1× reaction buffer (ThermoFisher Scientific, USA) and incubated at room temperature for different periods (1-10 hours).

*1.4 In vitro gliding assay of amplified DNA conjugated microtubules on a kinesin-coated glass substrate*

The flow cell was first plasma treated for 3 min by a plasma etcher (SEDE-GE; Meiwafosis) to make it hydrophilic. Then, 5 μL casein buffer (BRB80 buffer supplemented with 0.5 mg/mL casein) was applied on the flow cell. After incubating for 3 min, the flow cell was washed with 10 μL of motility buffer. Then, 5 μL of 800 nM kinesin solution (~80 mM PIPES, 1 mM EGTA, 1 mM MgCl_2_, 0.5 mg/mL casein, 1 mM DTT, 10 μM paclitaxel; pH 6.8) was introduced and incubated for 5 min. The flow cell was washed with 10 μL of motility buffer. Next, 5 μL of microtubule solution of prescribed concentration was introduced and incubated for 4 min, followed by washing with 10 μL of motility buffer. Then, 5 μL of 10 mM ATP buffer (~80 mM PIPES, 1 mM EGTA, 1 mM MgCl2, 0.5 mg/mL casein, 1 mM DTT, 10 μM paclitaxel; pH 6.8) was introduced into the flow cell. All the aforementioned experiments were performed at 25 ºC.

*1.5 Data analysis (Fractal dimension analysis and node analysis)*

To perform the fractal dimension analysis, representative fluorescence images of DNA networks were analyzed with different fields of view, by using Image J plugin “Fraclac”. Fractal dimension of DNA network was determined from the 2-D fluorescence image. The fluorescence image was then converted to binary image by using ImageJ. After that ImageJ plugin “Fraclac” box counting method is used to get the slope from the log-log plot of number Vs size of the boxes. This slope is considered as the fractal dimension of the network. For the image analysis, FD was calculated for 100 values of scale size (box size) ranging from 5 pixel (0.55 µm) to 400 pixel (44 µm). Total image size was taken 942×942 pixel (103.62 µm).

Node and connection analysis was performed using “StructuralGT” 3rd party software produced by Drew Vecchio and Samuel Mahler^39^. StructuralGT is a python-based software which uses graph theory to describe network patterns. Images for analysis were 8-bit 700x700 pixels and automatically converted to binary images in the software by Otsu automatic thresholding. Gaussian blur was applied to images to reduce noise.

In all image analyses, thresholding was repeated over a range of approximately ±10% of the selected value to ensure that the results were not sensitive to over- or under-thresholding.

# 2. Mathematical Model


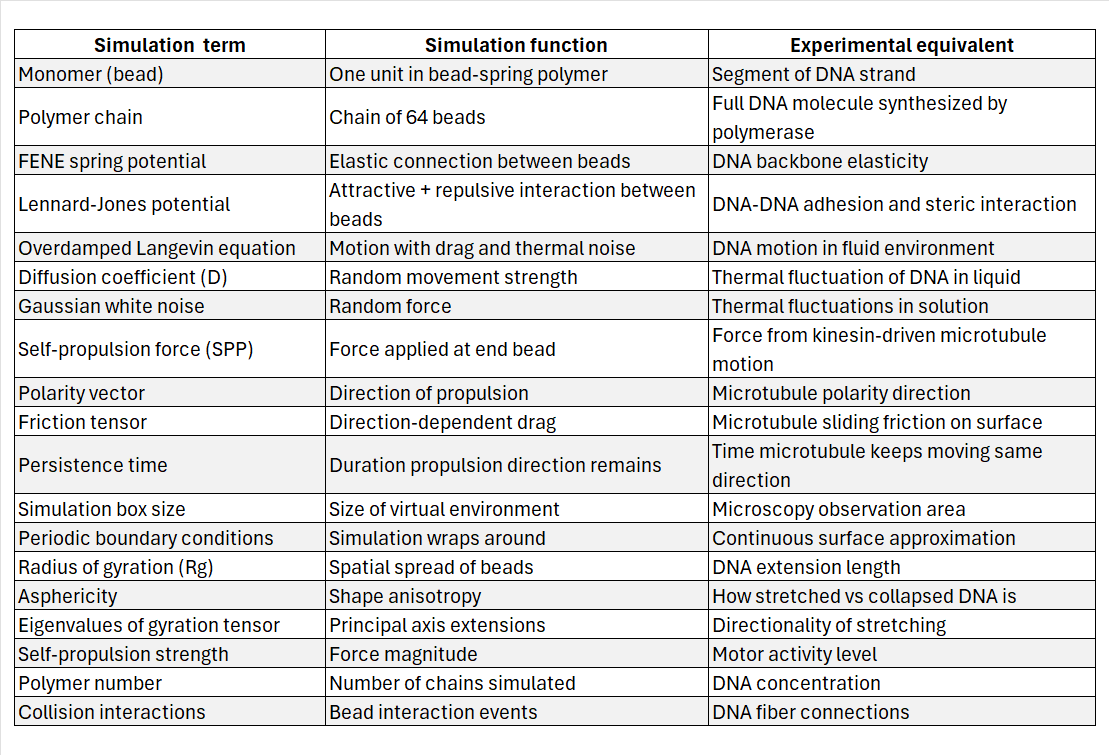


Table S1

In the in vitro experiment, each DNA strand is attached to a microtubule at one end, and that DNA-tethered microtubule glides over kinesin motors connected to a glass surface. The motor activity of kinesin propels the microtubule along its polarity direction as if each microtubule moves on the substrate spontaneously. DNA is carried by such a propelled microtubule. When such DNA polymers cross each other, they adhere to each other and get stretched due to the propulsion of the microtubule at its ends. Such stretched DNAs can again adhere to another DNA. Eventually, DNAs sometimes form a 2D DNA network structure.

To simulate this situation computationally, we mathematically model this system as follows. We consider a DNA polymer consisting of 64 monomers (represented as beads), consequtively connected by finitely extensible elastic springs. Each monomer represents a part of the DNA strand (coarse-grained model). To mimic the microtubule’s gliding motion, we apply self-propulsion force at the end of the DNA polymer. In addition, we assume the interaction between the monomers to phenomenologically mimic the tendency that the segments of DNA exclude each other by steric effect when they are too close but adhere to each other when they are located within an intermediate proximity. The mathematical details are provided in what follows.

The equation of motion that describes the dynamics of this stochastic system of $N$ beads (monomers) in a spring ball model, with position coordinates $\mathbf{r}_{\mathbf{i}}$ ($i=1,2,3,...,N$) in 2 dimensions, is given by the overdamped Langevin equation,

$\frac{d\mathbf{r}_{\mathbf{i}}}{dt}=-\frac{D_{T}}{k_{B}T}\frac{dU\left( \left\{ \mathbf{r}_{\mathbf{i}} \right\} \right)}{d\mathbf{r}_{\mathbf{i}}}+\sqrt{2D_{T}} \boldsymbol{\eta}_{\mathbf{i}}\left( t \right)$ (1)

where $D_{T}$ is the translational diffusion coefficient, $k_{B}$ is the Boltzmann constant, $T$ is the temperature, $\boldsymbol{\eta}_{\mathbf{i}}(t)$ is the Gaussian white noise with $<\boldsymbol{\eta}_{\mathbf{i}}(t)>=0$ and $<\boldsymbol{\eta}_{\mathbf{i}}\left( t \right)\cdot\boldsymbol{\eta}_{\mathbf{j}}(t')>=\delta_{ij}\delta_{\alpha\beta}\delta(t-t')$, with the greek letters representing the cartesian components.

For this coarse-grained polymer model, the potential energy $U\mathbf{(}\boldsymbol{\{}\mathbf{r}_{\mathbf{i}}\boldsymbol{\}}\mathbf{)}$ includes the contribution from $U_{\mathrm{LJ}}\left( \boldsymbol{\{}\mathbf{r}_{\mathbf{i}}\boldsymbol{\}} \right)$ representing interactions among all the bead-pairs­, bondend or non-bonded and the bond stretching part, $U_{\mathrm{FENE}}\left( \boldsymbol{\{}\mathbf{r}_{\mathbf{i}}\boldsymbol{\}} \right)$**,** chosen to a finitely extensible nonlinear elastic potential; $U\left( \boldsymbol{\{}\mathbf{r}_{\mathbf{i}}\boldsymbol{\}} \right)\mathbf{=}U_{\mathrm{LJ}}\left( \boldsymbol{\{}\mathbf{r}_{\mathbf{i}}\boldsymbol{\}} \right)\mathbf{+}U_{\mathrm{FENE}}\left( \boldsymbol{\{}\mathbf{r}_{\mathbf{i}}\boldsymbol{\}} \right)$. The FENE potential connects the beads into the polymer chains. The Lennard-Jones potential among the monomers is

$U_{\text{LJ}}(\boldsymbol{\{}\mathbf{r}_{\mathbf{i}}\boldsymbol{\}})=4\epsilon_{LJ}\sum_{i=1}^{N-1} \sum_{j=i+1}^{N} \left[ \left( \frac{\sigma}{{\Delta r}_{i,j}} \right)^{12}-\left( \frac{\sigma}{{\Delta r}_{i,j}} \right)^{6} \right]$ (2)

where $\epsilon$ is the strength of the interaction between two monomers $i$ and $j$ separated by distance ${\Delta r}_{i,j}\equiv\left| \mathbf{r}_{\mathbf{i}}-\mathbf{r}_{\mathbf{j}} \right|$ and $\sigma$ indicates the size of the monomer. The FENE potential is given by:

$U_{\text{FENE}}(\boldsymbol{\{}\mathbf{r}_{\mathbf{i}}\boldsymbol{\}})=-\frac{1}{2}kR_{FENE}^{2}\sum_{i=1}^{N-1} \text{ln}\left( 1-\left( \frac{{\Delta r}_{i,i+1}}{R_{FENE}} \right)^{2} \right)$ (3)

where $k$ is the spring constant, and $R_{FENE}$ is the maximum bond length of connectivity between two monomers.

Additionally, we have also applied a self-propulsion force at the end monomer of each DNA polymer, which models the force provided by the microtubule propelled by kinesin. The dynamics of this system are governed by the overdamped Langevin equations:

$\boldsymbol{\Theta}\frac{d\mathbf{r}_{\mathbf{j}}}{dt}=-\frac{1}{\zeta_{\parallel}}\frac{dU\mathbf{(}\mathbf{r}_{\mathbf{j}}\mathbf{)}}{d\mathbf{r}_{\mathbf{j}}}+v_{0}\mathbf{q}_{j}$ (4)

$\frac{d\theta_{j}}{dt}=\xi_{j}(t)$ (5)

Equations 4 and 5 represent the equation of motion for the last bead of each polymer where we assume that the bead acts as a self-propelled particle with a polarity. The 2nd term on the right-hand side of equation 4 is the self-propulsion force with a polarity vector $\mathbf{q}_{i}=(\cos\theta_{i},\sin\theta_{i})$. The parameter $v_{0}$sets the strength of the self-propulsion force. In the main text, we refer to this parameter as spp to emphasize its connection to self-propulsion. In the Supplementary Information, we use $v_{0}$​ and spp interchangeably. The term $\boldsymbol{\xi}_{\mathbf{i}}$ in the dynamics of a polarity direction is a white Gaussian noise satisfying $<\boldsymbol{\xi}_{\mathbf{i}}(t)>=0$ and $<\boldsymbol{\xi}_{\mathbf{i}}(t)\boldsymbol{\xi}_{\mathbf{j}}(t')>=2D\delta_{ij}\delta(t-t')$ with the noise strength $D$.This noise strength, $D$, has the dimension of inverse of time, and $D^{-1}$ indicates the persistence time of the polarity direction in its self-propulsion dynamics. We introduce an anisotropic friction tensor $\boldsymbol{\Theta}$ in order to realize different frictional forces along different directions assuming that a microtubule is hard to be glided transversally when it is collided by another microtubule from its side. We have defined $\boldsymbol{\Theta}(\mathbf{q}_{j})=\hat{\mathbf{q}_{j}}\bigotimes\hat{\mathbf{q}_{j}}+R_{\zeta}^{-1}(\mathbf{I}-\hat{\mathbf{q}_{j}}\bigotimes\hat{\mathbf{q}_{j}})$ in the equation of motion which allows the colliding DNA polymer to either slide through each other or stop moving, meanwhile the collided polymer does not move perpendicular to the angle of its polarity. Here $\hat{\mathbf{q}_{j}}=\frac{\mathbf{q}_{j}}{|\mathbf{q}_{j}|}$ is the unit vector in the polarity direction, $R_{\zeta}=\frac{\zeta_{||}}{\zeta_{\perp}}$ is the ratio of the parallel and perpendicular components of the friction coefficient, $\mathbf{I}$ is the identity matrix. $\zeta_{||}$ is the friction along the direction parallel to its polarity, $\hat{\mathbf{q}_{j}}$, whereas $\zeta_{\perp}$is the friction along the perpendicular direction. We have chosen the value of $R_{\zeta}$ such that the DNA polymers do not deflect much after collision.

# 3. Simulation units

We solve the overdamped Langevin equation by integrating it and updating the position $\mathbf{r}_{\mathbf{i}}$ at each time step. We simulate the cases with 1, 4 and 8 DNA polymers. Each polymer consisting of 64 monomers. The simulation box length is $L=24$ and periodic boundary conditions are applied in both $x$ and $y$ directions. We did this simulation by taking the simulation parameters in reduced units as follows: We set $k_{B}T$ to be the unit of energy, which is implemented just by using $k_{B}T=1$ in the simulations. The unit of length is set by the diameter of the monomer as $l\equiv3\times2^{1/6}\sigma=1$. The rotational diffusivity $D_{R}$=1 is fixed to give the inverse of the time unit. Considering a spherical Brownian particle and Stoke’s law, we can obtain the unit of the translational diffusivity $D_{T}=1/27$ simulation units. The other chosen simulation parameters are $\epsilon_{LJ}=2.0$, $R_{\zeta}=0.00001$. All the parameters are chosen such that we can observe the phenomena similar to the experiment and achieve the simulation model which helps in understanding the system better. We numerically solve the equation of motions with discretized time steps $\Delta t=0.0001$ with total iterations of $1\times{10}^{7}$. The first $2\times{10}^{6}$ steps are used for the thermal equilibrium of the system. The remaining steps are used to record the trajectory of the monomers. From these trajectories, we study the dynamic and structural properties of the polymer model. We systematically varied the self-propulsion strength $v_{0}=0,0.1,0.2,0.25,0.3,0.35,0.4,0.45,0.5$.

# 4. 2D Gyration tensor

To quantify the shape more precisely, we calculate the two-dimensional gyration tensor of the DNA polymer. It explains the spatial distribution of monomers of a polymer around the center of mass.

The gyration tensor $\mathbf{S}$ in two dimensions is given by:

$\mathbf{S}=\left( \begin{matrix} S_{xx} & S_{xy} \\ S_{yx} & S_{yy} \end{matrix} \right)$ (6)

where the components are defined as:

$S_{xx}=\frac{1}{N}\sum_{i=1}^{N} (x_{i}-x_{\text{cm}})^{2}$ (7)

$S_{yy}=\frac{1}{N}\sum_{i=1}^{N} (y_{i}-y_{\text{cm}})^{2}$ (8)

$S_{xy}=S_{yx}=\frac{1}{N}\sum_{i=1}^{N} (x_{i}-x_{\text{cm}})(y_{i}-y_{\text{cm}})$ (9)

Here, $(x_{i},y_{i})$ are the coordinates of the $i$-th monomer, and $(x_{\text{cm}},y_{\text{cm}})$ is the center of mass of the polymer. $N$ is the total number of monomers.

# Eigenvalues of the Gyration Tensor

The eigenvalues $\lambda_{1}$ and $\lambda_{2}$ of the gyration tensor are obtained by solving the characteristic equation:

$\text{det}(\mathbf{S}-\lambda\mathbf{I})=0$ (10)

The eigenvalues are given by:

$\lambda_{1,2}=\frac{1}{2}[(S_{xx}+S_{yy})\pm\sqrt{\left( S_{xx}+S_{yy} \right)^{2}-4(S_{xx}S_{yy}-S_{xy}^{2}})]$ (11)

where $\lambda_{1}$ is the larger eigenvalue and $\lambda_{2}$ is the smaller eigenvalue. These two eigenvalues indicate the distribution of monomers along two principal axes of the gyration tensor. $\lambda_{1}$(the largest eigenvalue) represents the spatial extent of the polymer along the major axis and $\lambda_{2}$(the smallest eigenvalue) represents the spatial extent along the minor axis. Analyzing the radius of gyration and shape anisotropy in terms of the principal axes allows insight into the polymer’s alignment and deformation in 2D space.

# Radius of Gyration

By calculating the $R_{g}$ values of each simulation case, we can get an idea of the polymer chain’s conformation, the distribution of the polymer’s mass around the center of mass, and whether it is compact or stretched. The radius of gyration is a good measure of a polymer chain in the sense that it gives a quantitative idea of how much the polymer chain is extended around its center of mass. From the gyration matrix calculation, we can define the radius of gyration as

$$R_{g}=\sqrt{\lambda_{1}+\lambda_{2}}$$

# Anisotropy Ratio

The anisotropy ratio is defined as the ratio of the eigenvalues:

$$\text{Anisotropy Ratio}=\frac{\lambda_{2}}{\lambda_{1}}$$

This parameter informs the extension of the polymer chain along a particular direction of the principal axis.

# Asphericity or shape anisotropy

Asphericity or shape anisotropy of the polymer is a measure of how much the shape of the polymer deviates from being circular, and it is defined as:

$$\text{Asphericity}=\frac{(\lambda_{1}-\lambda_{2})^{2}}{(\lambda_{1}+\lambda_{2})^{2}}$$

This parameter is bounded between 0 (perfectly isotropic) and 1 (highly anisotropic). When the polymer is perfectly circular (which means it is highly compact and both the eigenvalues are equal in number indicating equal extension in both directions) the asphericity gives a 0 value; so all the near-zero values indicate the shape of the polymer changing from compact to an extended chain. Meanwhile, if this number is 1 (which means the polymer is completely extended in one of the principal axis directions) or near 1 values it shows that the polymer is stretched. This parameter helps to recognize whether there is a transition of the polymer when the self-propulsion increases, hence quantifying the conformation of the polymer chain.

# 5. Simulation results

## 5.1 2D configuration of DNA at different Self Propulsion Force

The strength of self-propulsion force (SPP, defined as $v_{0}$, from equation 4) is an important control parameter in our simulation model. To achieve a stable 2d network structure of DNA, we vary the spp values by keeping other parameters constant. We study the structural changes of the DNA polymer from collapsed to stretched polymer state as they collide with each other due to the motion of gliding microtubules. **Figure S**[**1**](#fig:snap) shows the structure of 1,4 and 8 DNAs concerning change in the strength of self-propulsion force values. In 1 DNA case, we observe that the DNA remains collapsed up to spp=0.3 and starts to stretch above this value. This is due to the high self-propulsion force at the end of DNA which dominates over the monomer-monomer affinity. In 4 DNA and 8 DNA cases, the DNA starts with a collapsed state at spp=0. With the increase in spp value, the DNA gets uncoiled after the collision with each other above spp=0.2. There is an apparent crossover around spp=0.2. We observe a network-like structure at higher spp values and speculate that with higher number density we might get a stable network structure.


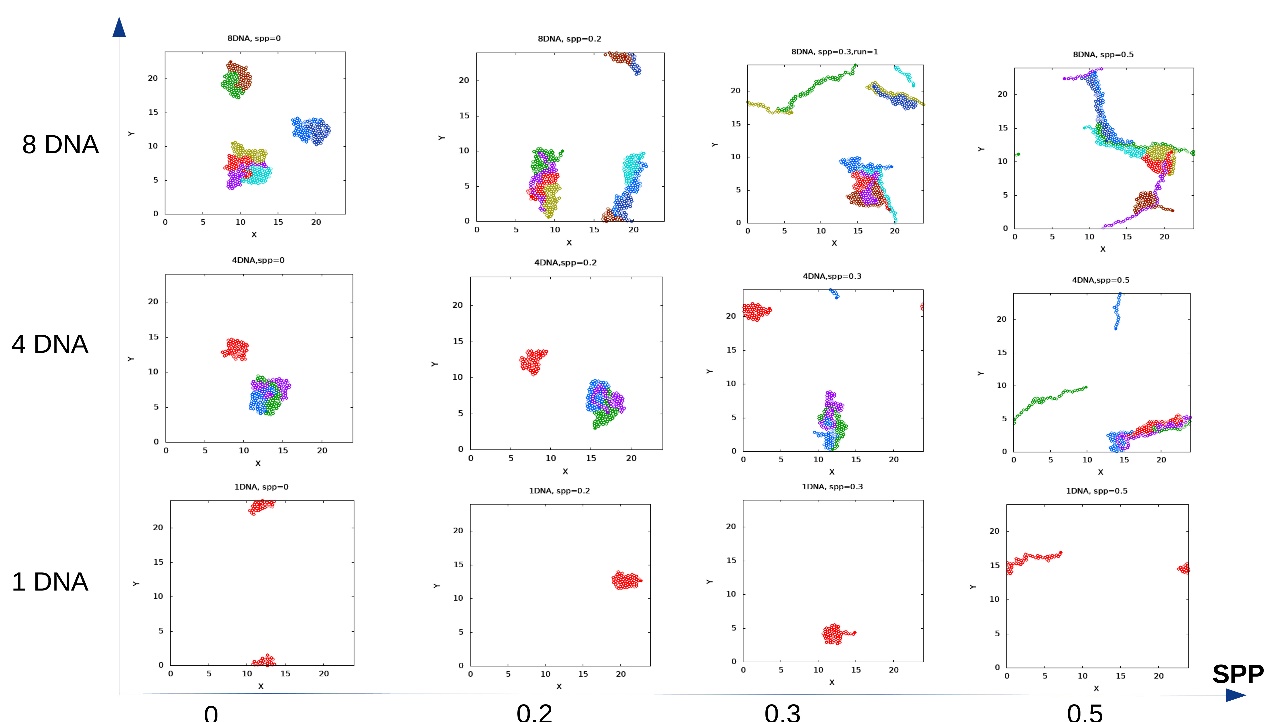


**Figure S1.** 2D configuration of the 1,4,8 DNA at different spp=0,0.2,0.3,0.5

## 5.2 Quantitative analysis

**
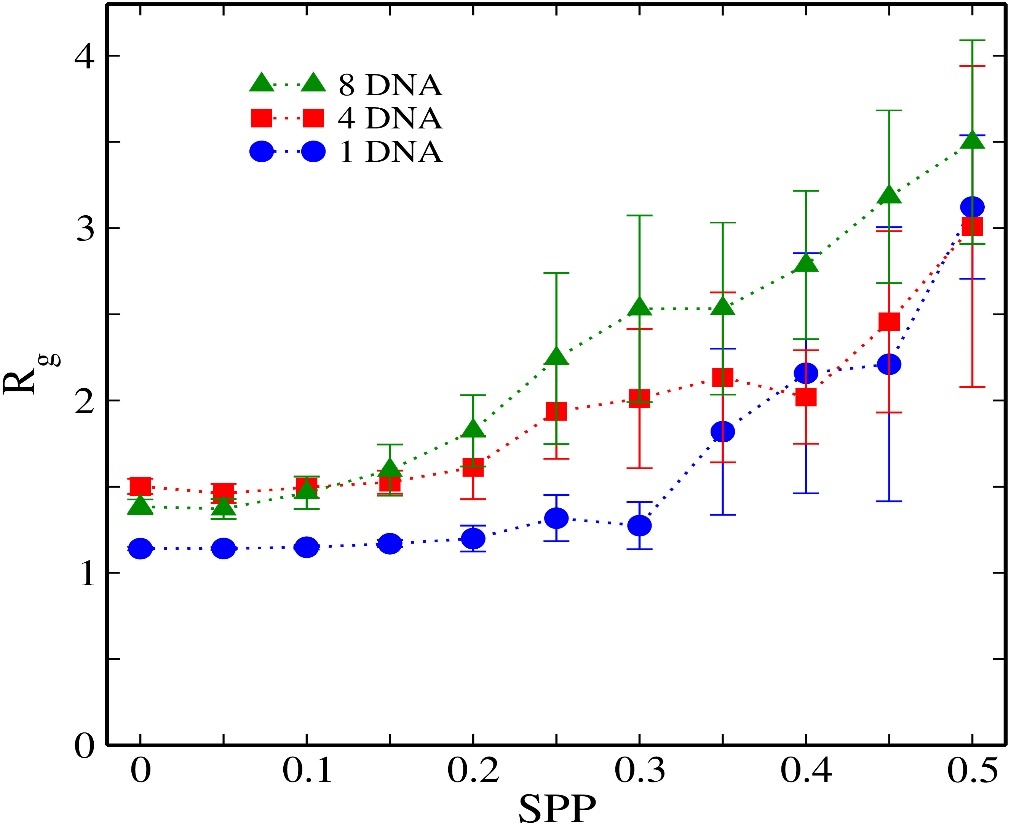
**In **Figure S2**, we present data for the average $R_{g}$ values (averaged over 10 samples and many snapshots) against different spp values for all the DNA systems studied. We observe an increase in the $R_{g}$ values above spp=0.2 for all the DNA systems indicating the uncoiling of the DNA polymer and formation of a network-like structure. We can observe that for the multiple DNA case, the $R_{g}$ value starts to increase for a comparatively smaller value of spp (<0.2) than the single DNA case. However, there is a crossover around spp=0.2 and we observe higher $R_{g}$ values for spp$>0.2$. This is because the intra-DNA interaction boosts the stretching of the DNA polymer when two of them collide with each other. The important point to note here is that at higher spp values the DNA can be extended even without the interaction among the DNA as seen in the single DNA case. This is due to the tug-of-war between the friction on all monomers and self-propulsion force at only on the end monomer. The regime, around spp=0.3, is the main regime of our interest in terms of experimental observation.

**Figure S2.** Average value of Radius of gyration of 1 (Circle), 4(Square), and 8(Triangle) DNA.


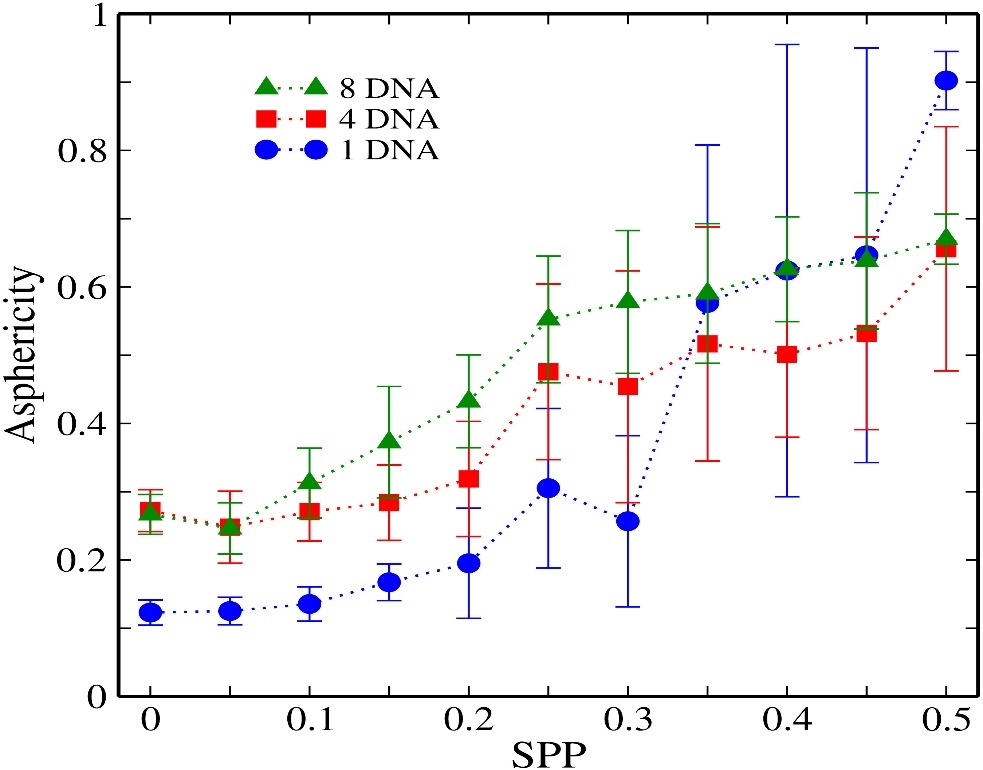
***Figure S3.*** *Average value of asphericity of 1 (Circle), 4(Square), and 8(Triangle) DNA.*

Asphericity or shape anisotropy is another parameter that is very useful to explain the polymeric system which is anisotropic in nature and measures the conformation of the polymer chain during its course of motion. In **Figure S**[**3,**](#fig:snap) we plotted the different values of asphericity averaged over many realizations against the spp value for all three cases of 1,4 and 8 DNA. The low self-propulsion force does not overcome the monomer-monomer affinity; hence, the DNA polymer remains more or less in a collapsed state. This can be observed in the curve below spp=0.2 which shows low values (near to zero) in the asphericity. The more the extension of the polymer chain the more the asphericity value which just explains that the chain is extended more in one of the spatial directions compared to the other. With the gradual increase in the self-propulsion strength the asphericity increases. An absolute linear stretch along one axis will give the asphericity as 1; however, in our case, though the polymer chain is extended it still does not perfectly stretch to a linear structure.

At $\mathrm{spp}>0.3$, even the 1 DNA case shows higher values of $R_{g}$ and asphericity. This is the natural consequence of the balance of the friction on all the monomers in the polymer and self-propelled force only at the end monomer, by which the single polymer is stretched. This is not what we observe in our experimental condition. Therefore, in the main text, we focus on $\mathrm{spp}\leq0.3$, where we can avoid this effect.

# 6. Summary

The DNA-Microtubule-kinesin system was studied in the in vitro experiment where it was observed that the DNA forms a stable 2D network structure. We carried out the in-silico study of this system by solving the overdamped Langevin equation of a polymer chain with the spring-bead model; each DNA polymer is applied with a self-propulsion force at the end of the bead to mimic the microtubule motion. We observe that the spp value is an important control parameter and we systematically studied the structural changes of the polymer chain depending on the spp values. The radius of gyration calculation reveals a crossover near spp=0.2 where the collapsed DNA starts to uncoil and eventually forms an apparent network structure in the presence of multiple DNA in the system. Similarly, asphericity confirms the stretching of the DNA polymer and hence the starting of network formation at higher spp values. We can predict that achieving a stable network structure will be feasible at a higher number of DNA. This numerical study provides a theoretical explanation of the study done in the *in-vitro* experiment.

**Supplemental Movies**

**Movie S1: Microtubule motion**

Confocal time series of motile microtubules (red) moving across a kinesin modified surface in the presence of ATP. Images captured at 3.3 seconds per frame, movie displayed at 7 frames per seconds. Red and green channels are merged.

**Movie S2: DNA stretched between microtubule and surface**

Confocal time series of motile microtubules (red) stretching tethered DNA (green) between the surface and the microtubules. Images captured at 3.0 seconds per frame, movie displayed at 7 frames per seconds. Red and green channels are merged.

**Movie S3: DNA stretched between two microtubules**

Confocal time series of DNA (green) being stretched between two motile microtubules (red) moving in opposite directions. Images captured at 3.0 seconds per frame, movie displayed at 7 frames per seconds. Red and green channels are merged.

**Movie S4: DNA network formation**

Confocal time series of DNA (green) stretched network formation over time. Images captured at 19.33 seconds per frame, movie displayed at 7 frames per seconds. Red and green channels are merged.

**Movie S5:** **DNA stretch into network by microtubule action**

Confocal time series showing DNA (green) being stretched into a connecting network by the action of motile microtubules (red). Images captured at 3.0 seconds per frame, movie displayed at 7 frames per seconds. Red and green channels are merged.

**Movie S6:** **Microtubule motion over DNA**

Confocal time series of microtubules (red) moving across a stretched DNA filament (green). Images captured at 3.3 seconds per frame, movie displayed at 7 frames per seconds. Red and green channels are merged.

**Movie S7:** **DNA addition to the surface by microtubules**

Confocal time series of DNA (green) coming to the surface and being stretched out by microtubule (red) motion. Images captured at 3.3 seconds per frame, movie displayed at 7 frames per seconds. This movie consists of green channel, red channel and red and green merged channels of the same event, shown sequentially to facilitate understanding.

**Movie S8:** **DNA bundling and thickening**

Confocal time series of several neighboring thin DNA stretched fibers interacting and apparent merging into thicker DNA fibers. Images captured at 3.0 seconds per frame, movie displayed at 7 frames per seconds. This movie consists of green channel only.

**Movie S9:** **1 Simulated** **DNA analogue**

Example of a simulated movie of a singular DNA analogue under a self-propulsion force of 0.3

**Movie S10:** **8 Simulated DNA analogues**

Example of a simulated movie of a 8 DNA analogues under a self-propulsion force of 0.3
